# On the origin of second harmonic generation in silicon waveguides with silicon nitride cladding

**DOI:** 10.1038/s41598-018-37660-x

**Published:** 2019-01-31

**Authors:** Claudio Castellan, Alessandro Trenti, Chiara Vecchi, Alessandro Marchesini, Mattia Mancinelli, Mher Ghulinyan, Georg Pucker, Lorenzo Pavesi

**Affiliations:** 1 Nanoscience Laboratory, Department of Physics, University of Trento, via Sommarive 14, Trento, 38123 Italy; 2 Centre for Materials and Microsystems, Fondazione Bruno Kessler, via Sommarive 18, Trento, 38123 Italy; 3 Present Address: Vienna Center for Quantum Science and Technology (VCQ), Faculty of Physics, University of Vienna, Boltzmanngasse 5, 1090 Vienna, Austria

## Abstract

Strained silicon waveguides have been proposed to break the silicon centrosymmetry, which inhibits second-order nonlinearities. Even if electro-optic effect and second harmonic generation (SHG) were measured, the published results presented plenty of ambiguities due to the concurrence of different effects affecting the process. In this work, the origin of SHG in a silicon waveguide strained by a silicon nitride cladding is investigated in detail. From the measured SHG efficiencies, an effective second-order nonlinear susceptibility of ~0.5 pmV^−1^ is extracted. To evidence the role of strain, SHG is studied under an external mechanical load, demonstrating no significant dependence on the applied stress. On the contrary, a 254 nm ultraviolet (UV) exposure of the strained silicon waveguide suppresses completely the SHG signal. Since UV irradiation is known to passivate charged defects accumulated in the silicon nitride cladding, this measurement evidences the crucial role of charged centers. In fact, charged defects cause an electric field in the waveguide that via the third order silicon nonlinearity induces the SHG. This conclusion is supported by numerical simulations, which accurately model the experimental results.

## Introduction

Integrating optical components on the silicon platform is appealing due to its compatibility with the complementary metal-oxide-semiconductor (CMOS) technology. This offers interesting perspectives for the mass-production of integrated photonic circuits^1^. Many devices which are suited for diverse applications from bio-sensing to optical routing were demonstrated^2,3^. Our attention is specifically devoted to nonlinear optics in the silicon photonics platform since the small waveguide size and the large modal confinement favor a high power density for a low input optical power^4^. This leads to the realization of integrated photonic devices, e.g., for wavelength conversion or generation of quantum states of light^5,6^. These exploit third order nonlinearities, since in the dipole approximation second order nonlinearities are inhibited in bulk silicon due to its centrosymmetric crystalline structure^7^. However, second order nonlinearities, which require a lower threshold power than third order effects, would be advantageous for, e.g., high frequency electro-optical modulation or spontaneous parametric down-conversion. Therefore, many efforts have been paid to induce second order nonlinearities in silicon. The most successful works were based on strained silicon, where a stressing over-layer deposited on the top of the silicon waveguide breaks the silicon bulk centrosymmetry^8^. The first experiment reported on the observation of a DC electro-optic effect in waveguides stressed by a silicon nitride (SiN) over-layer^9^. This observation was followed by many others, and strain-induced second order nonlinear coefficients *χ*^*(2)*^ up to 340 pmV^−1^ were reported^10,11^. Other works, some of which were based on high frequency measurements, questioned the interpretation of these experiments. They demonstrated that frozen charges at the Si/SiN interface determine an abundance of free-carriers in the waveguide, causing a large DC electro-optic effect due to free carrier dispersion^12–15^. In particular, an upper limit of 8 pmV^−1^ to the value of the strain-induced *χ*^*(2)*^ was set^13^. In a very recent work^16^, a high-frequency experiment was able to demonstrate an electro-optic modulation that is ascribed to a strain-induced *χ*^*(2)*^ of the order of 1.8 pm/V. The coefficients connecting strain gradients to the strain-induced *χ*^*(2)*^ were fitted from the experiment according to the theoretical model proposed in^17^. Still no proof that the data are related to the Pockels effect^10^ and not to the Kerr effect^18^ is clearly given. Other early works were about second harmonic generation (SHG) in strained silicon waveguides^19^. By using pump wavelengths above 2 μm, SH was generated in multimodal waveguides. A *χ*^*(2)*^ as large as (40 ± 30) pmV^−1^ was deduced by the generation efficiency without accounting for any phase-matching mechanism. Subsequently, SHG experiments performed on differently stressed waveguides yield indication that not only strain but also charged defects in the cladding could explain the SHG signal^20^. In fact, charged defects could introduce a DC field *E*_*DC*_ that couples to the pump photons through the third order nonlinear coefficient *χ*^*(3)*^. This generates an effective nonlinearity ${\chi }_{EFISH}^{(2)}=3{\chi }^{(3)}{E}_{DC}$, which is known as the electric-field-induced SH (EFISH). Finally, SiN possesses a non-negligible *χ*^*(2)*^ ^21,22^. So, the observed SHG could be due to the evanescent field of the pump mode that extends in the cladding.

The complexity of the materials, where strain, stress, interfaces and residual doping all play a role, impedes a clear disentanglement of these possible contributions and of their relative importance. This limits the development of second order nonlinear devices based on strained silicon. Therefore, the aim of this work is to confirm the observation of SHG in suitably designed waveguides and to clarify the origin of the nonlinearity. Here, we report on the quantitative measurements of the SHG efficiency in phase-matched silicon waveguides covered by a stressing SiN cladding. Using a pulse propagation modeling, we estimate a *χ*^*(2)*^ of 0.5 ± 0.1 pmV^**−**1^. To clarify its origin, we use a screw-equipped sample holder, which allows inducing a tunable strain on the waveguides. Surprisingly, this does not affect the SHG efficiency. On the other hand, the SHG signal is suppressed when the strained silicon waveguides are irradiated by ultraviolet (UV) light to passivate the charged defects^23^. These two observations allow us to conclude that, within our experimental resolution, the measured SHG signal is entirely due to EFISH caused by the charged defects. Assuming that the strain-induced nonlinearity is below the noise level of our measurement, we set an upper limit to the strain-induced *χ*^*(2)*^ at about 0.05 pmV^**−**1^.

## Second Harmonic Generation Experiment

### Experimental set-up

**Figure 1:**
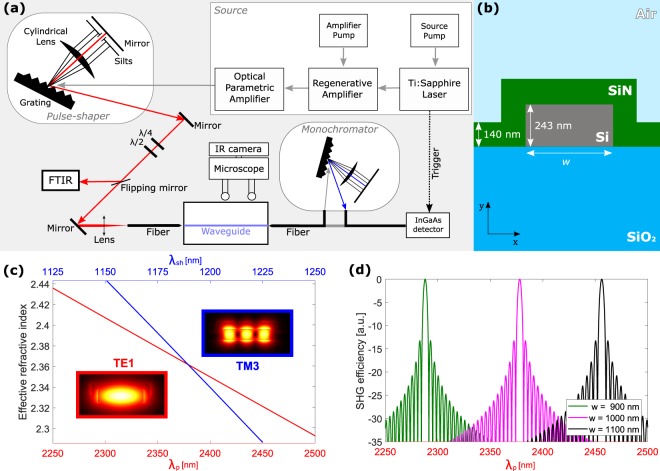
(**a**) Sketch of the experimental set-up. (**b**) Waveguide cross section with nominal dimensions. The width *w* is engineered for each modal combination to achieve phase-matching. (**c**) Effective refractive index dispersions for the TE1 mode at pump wavelengths (red line) and for the TM3 mode at SHG wavelengths (blue line). In the simulation *w* = 1 µm. The insets show the mode profiles for the pump and SHG modes. (**d**) Dependence of the SHG efficiency (in normalized dB scale) on pump wavelength for the different waveguide widths reported in the inset (combination TE1–TM3).

The experimental setup is similar to the one described in^24,25^ and is shown in Fig. 1(a). A regenerative amplified Ti:Sapphire laser pumps an optical parametric amplifier to obtain high power optical pulses with tunable wavelengths above 2.2 µm, 1 kHz repetition rate and 35 fs pulse duration. The use of a wavelength above 2.2 µm has two reasons. First, it prevents from two-photon absorption (TPA) within silicon. TPA competes with SHG, limiting the maximum achievable pump peak power in the waveguide and generating free carriers, which cause losses both at the pump and the SH wavelengths^26^. Second, with a >2.2 µm pump, SH is generated above 1.1 µm, where silicon is transparent. A pulse shaping stage allows to lengthen the pump pulse duration to up to 5 ps, which limits the peak power on the waveguide facet and reduces walk-off effects in the waveguide^27^. A polarization stage controls the pulse polarization, while a Fourier transform infrared spectrometer (FTIR) measures its spectrum. Using a tapered lensed fiber, an average power of up to 100 nW is coupled into the waveguide. The waveguide is mounted on a screw-equipped sample holder like the one described in^13,28^. This special holder is used to apply an external and tunable mechanical load to the waveguide. A second lensed fiber collects the SHG signal, which is detected by an InGaAs single photon avalanche detector (SPAD). The pump pulse triggers the SPAD, opening a detection window of 2.5 ns and setting the detection limit to about 0.004 fW for 1s of integration time. A scanning monochromator positioned between the fiber and the SPAD allows spectral analysis of the SHG signal.

### Waveguide engineering

The devices are fabricated on a 6′ silicon-on-insulator (SOI) wafer, with a 243 nm thick device layer and a 3 μm thick buried-oxide (BOX). The waveguides are defined by a 365 nm UV lithography and realized by reactive ion etching. A 140 nm thick SiN cladding layer is conformally deposited via low-pressure chemical vapor deposition (LPCVD), causing a measured tensile stress of 1.25 GPa in the silicon nitride cladding. Different silicon waveguide widths are used. The cross section of the waveguide is sketched in Fig. 1(b).

For SHG, the phase-matching condition requires the pump (*n*_*p*_) and the SH (*n*_*sh*_) effective refractive indices to be equal (*n*_*p*_ = *n*_*sh*_). Due to the index dispersion, the pump and the SH signal propagating in the same modal order do not have equal effective indices. Therefore, we use different modes to get the phase-matching: the pump and the SH signals propagate in different modes which have the same effective modal indices^21,29^. We investigate two modal combinations: both have the pump in the fundamental transverse electric (TE1) mode, while, in the first case, SH is generated on the third order transverse magnetic (TM3) mode and, in the second case, SH is generated on the fifth order transverse magnetic (TM5) mode. Phase-matching is achieved by changing the waveguide width *w* (the first type has *w* ~ 1 µm, the second *w* ~ 2.3 µm). Figure 1(c) reports the simulated dependence of the effective refractive index versus the pump wavelength *λ*_*p*_ for the TE1 mode and the SH wavelength *λ*_*sh*_ for the TM3 mode in a 1 µm wide waveguide. At *λ*_*p*_ ~ 2378 nm, *n*_*p*_ = *n*_*sh*_, i.e. phase-matching is satisfied. The insets show the mode profiles for the pump and the SH waves. One can notice that, for equal parity modes the overlap integral is not negligible, which contributes to the generation efficiency. We perform these simulations using a finite element method (FEM) simulation software, taking material refractive index dispersions measured with ellipsometry^30^. SHG efficiency is proportional to sinc^*2*^*(ΔβL/2)*, being *L* the waveguide length and *Δβ* = *2β*_*p*_ − *β*_*sh*_ = *2π*(*n*_*p*_ − *n*_*sh*_)*/λ*_*sh*_ the phase mismatch coefficient^31^. In Fig. 1(d) we plot the SHG efficiency in a normalized dB scale for the TE1–TM3 combination as a function of *λ*_*p*_ and for different *w*, considering *L* = 2 mm. Phase-matching wavelength strongly depends on *w*, and larger *w* requires longer wavelengths.

### Second harmonic generation characterization

**Figure 2:**
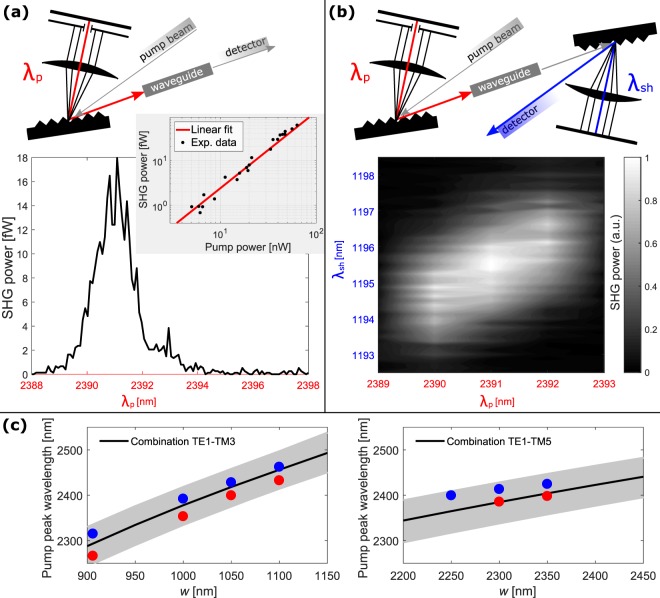
(**a**) Measured SHG power as a function of *λ*_*p*_. On the top: sketch of the setup used in this experiment. The waveguide is 4 mm long and 1050 nm wide. In the inset: SHG power dependence on the pump power. Results are fitted by a straight line in a log-log plot. (**b**) Spectral analysis of the generated signal performed using the set-up sketched on the top. (**c**) Dependence of the phase-matching pump wavelength on *w*. Data refer to the TE1–TM3 (left) and to the TE1–TM5 combination (right). Points with the same color refer to waveguides located nearby on the wafer. Black line is the behavior predicted by FEM simulations, while the gray area comes from simulations with 5% variations on *w*.

Figure 2(a) shows experimental SHG power as a function of *λ*_*p*_, i.e. the spectrum of the SHG efficiency. The measurement is performed on a 1050 nm wide 4 mm long waveguide, where phase-matching of the TE1–TM3 combination is expected. The SHG signal strongly depends on *λ*_*p*_, showing a peak at 2391 nm which corresponds to the phase-matching wavelength.

The attribution of the signal to SHG is also confirmed by the quadratic dependence of the SHG power on the pump power, shown in the inset of Fig. 2(a). In the log-log plot a slope of 1.81 ± 0.12 is observed, which almost matches the theoretical expectation of 2^7^. The on-chip pump power is estimated from the incident power knowing the in-coupling losses, which are determined via the cut-back method (9 dB per facet for *w* = 1 µm, and 4 dB per facet for *w* = 2.3 µm)^32^. The on-chip SHG power is determined from the SPAD signal knowing its responsivity at the SH wavelength and the out-coupling losses. These are evaluated analytically from the field profile of the fiber and of the involved modes (15.5 dB per facet for the TM3 mode in the *w* = 1 µm waveguide, and 21.7 dB per facet for the TM5 mode in the *w* = 2.3 µm waveguide)^33^.

SHG is confirmed also by the spectral analysis of the generated signal, shown in Fig. 2(b), where both the pump and the SHG wavelengths are scanned. A clear SHG spectrum centered at one-half of pump wavelength is observed.

Figure 2(c) reports the phase-matching pump wavelength as a function of *w*, for the TE1–TM3 (left) and for the TE1–TM5 (right) combinations. Measurements referred to the same position in the wafer (same color) show a monotonous dependence on *w*. The trend agrees with the one predicted by simulations, reported as a straight line. However, data show an offset with respect to the theory. This offset is different between nominally equal waveguides located in different positions of the wafer (different colors). We attribute this offset to local variations of *w* on the wafer. This is evidenced by the gray area in the figure, corresponding to simulations with 5% variations on *w* (according to typical fabrication uncertainties). Experimental results fall in the area predicted by simulations. Moreover, waveguides positioned in the same wafer position show almost the same shift with respect to the theory, demonstrating local uniformity of the waveguide geometry on the short-scale distance.

### Theoretical model

We estimate the strength of the non-linearity causing SHG by modelling the propagation of the pump and SHG pulses in the waveguide^4^. The model considers that, besides SHG, other processes affect the generation. Due to the short time duration of the pulses, dispersion is important. Moreover, due to the strong *χ*^*(3)*^ of silicon, also self-phase modulation (SPM) and cross-phase modulation (XPM) are significant. The model describes the temporal and spatial evolution of the dimensionless pump and SHG field amplitudes, *u*_*p*_ and *u*_*sh*_, related to the total optical power by *P*_*p*_ *|u*_*p*_*|*^*2*^ and *P*_*sh*_ *|u*_*sh*_*|*^*2*^. The model solves the following coupled equations^4^:

1a $$\begin{array}{rcl}\frac{d}{dz}{u}_{p}+\sum _{m\ge 1}\frac{{(i)}^{m-1}{\beta }_{m}}{m!}\frac{{d}^{m}}{d{t}^{m}}{u}_{p} & = & -\,\frac{{\alpha }_{p}}{2}{u}_{p}+i{\gamma }_{p}^{(3)}{P}_{p}{|{u}_{p}|}^{2}{u}_{p}\\  &  & +\,2i{\gamma }_{p}^{(2)\ast }\sqrt{{P}_{sh}}{u}_{sh}{u}_{p}^{\ast }\,\exp (\,-\,i{\rm{\Delta }}\beta z),\end{array}$$

1b $$\begin{array}{rcl}\frac{d}{dz}{u}_{sh}+\sum _{m\ge 1}\frac{{(i)}^{m-1}{\beta }_{m}}{m!}\frac{{d}^{m}}{d{t}^{m}}{u}_{sh} & = & -\,\frac{{\alpha }_{sh}}{2}{u}_{sh}+2i{\gamma }_{sh,p}^{(3)}{P}_{p}{|{u}_{p}|}^{2}{u}_{sh}\\  &  & +\,i{\gamma }_{sh}^{(2)}\frac{{P}_{p}}{\sqrt{{P}_{sh}}}{u}_{p}^{2}\,\exp (i{\rm{\Delta }}\beta z).\end{array}$$

On the left-hand side, spatial and temporal derivatives are present. The coefficients *β*_*m*_ are the Taylor expansion terms of the mode propagation constant *β*^4^. On the right-hand side there are a loss term (described by loss coefficients *α*_*p*_ and *α*_*sh*_), a phase-modulation term (related to the third order nonlinear coefficient *γ*^*(3)*^), and the SHG term (related to the second order nonlinear coefficient *γ*^*(2)*^). Since the SHG power is much weaker than the pump power, in the pump equation we consider only SPM and we neglect XPM, while in the SHG equation we consider only XPM. The XPM and SPM terms are proportional to ${\gamma }_{ij}^{(3)}$ and ${\gamma }_{i}^{(3)}$, defined as:

2 $${\gamma }_{ij}^{(3)}=\frac{3{\omega }_{i}{n}_{G,i}{n}_{G,j}}{4{\varepsilon }_{0}{A}_{0}{c}^{2}}{{\rm{\Gamma }}}_{ij}^{(3)},\,{\gamma }_{i}^{(3)}={\gamma }_{ii}^{(3)}.$$

The factor *ω*_*i*_ is the *i*-th pulse frequency, *n*_*G*,*i*_ the group index of the mode at frequency *ω*_*i*_, *A*_*0*_ the waveguide area and *c* the speed of light in vacuum. The overlap term ${{\rm{\Gamma }}}_{ij}^{(3)}$ is defined by:

3 $${{\rm{\Gamma }}}_{ij}^{(3)}={A}_{0}\tfrac{{\int }_{{A}_{\infty }}{E}^{\ast }({r}_{\perp },{\omega }_{j}){\chi }^{(3)}\therefore E({r}_{\perp },{\omega }_{i}){E}^{\ast }({r}_{\perp },{\omega }_{i})E({r}_{\perp },{\omega }_{j})dA}{({\int }_{{A}_{\infty }}n{({r}_{\perp },{\omega }_{i})}^{2}|E({r}_{\perp },{\omega }_{i}){|}^{2}dA)({\int }_{{A}_{\infty }}n{({r}_{\perp },{\omega }_{j})}^{2}|E({r}_{\perp },{\omega }_{j}){|}^{2}dA)},$$

where *χ*^*(3)*^ is the third order susceptibility tensor, *r*_*⊥*_ the coordinate in the waveguide cross-section plane, E(*r*_*⊥*_, *ω*_*i*_) the mode profile at frequency *ω*_*i*_ and *n(r*_*⊥*_, *ω*_*i*_*)* the refractive index at frequency *ω*_i_. Integrals are taken on the transverse plane *A*_*∞*_. In the SHG term ${\gamma }_{i}^{(2)}$ is given by:

4 $$\begin{array}{c}{\gamma }_{i}^{(2)}=\frac{{\omega }_{i}{n}_{G,p}\sqrt{{n}_{G,sh}}}{\sqrt{8{\varepsilon }_{0}{A}_{0}{c}^{3}}}{{\rm{\Gamma }}}^{(2)},\\ {{\rm{\Gamma }}}^{(2)}=\sqrt{{A}_{0}}{\textstyle \tfrac{{\int }_{{A}_{{\rm{\infty }}}}{E}^{\ast }({r}_{\perp },{\omega }_{p}){\chi }^{(2)}\,:\,E({r}_{\perp },{\omega }_{sh}){E}^{\ast }({r}_{\perp },{\omega }_{p})dA}{({\int }_{{A}_{{\rm{\infty }}}}n{({r}_{\perp },{\omega }_{p})}^{2}\,|E({r}_{\perp },{\omega }_{p}){|}^{2}dA){({\int }_{{A}_{{\rm{\infty }}}}n{({r}_{\perp },{\omega }_{sh})}^{2}|E({r}_{\perp },{\omega }_{sh}){|}^{2}dA)}^{1/2}}}.\end{array}$$

where *χ*^*(2)*^ is the second order susceptibility tensor. Note that *Γ*^*(2)*^ depends on both the pump and SH optical mode profiles, as already underlined.

We solve Equations (1a) and (1b) using the split-step method, dividing the spatial domain in small segments^34^. The initial condition is the temporal profile of the pump pulse (we use a Gaussian shape) and of the SH pulse (set to zero). In each spatial segment, the solution is evaluated in three steps. First, the equations are solved in the Fourier domain, considering only dispersion. Second, first step solutions are used as initial conditions to solve the equations with SPM, XPM and losses alone. Third, the equations are coupled via the SHG term. This procedure is then iterated until the end of the waveguide.

The unknown parameter of Equations (1a) and (1b) is the tensor *χ*^*(2)*^. Therefore, given the experimental SHG power, we aim to find *χ*^*(2)*^. However, since the spatial distribution of *χ*^*(2)*^ is unknown and is expected to be not constant, *χ*^*(2*)^ remains encoded within *Γ*^*(2)*^. So, *Γ*^*(2)*^ is the parameter that can be evaluated from the model. *Γ*^*(2)*^ in general is complex, so the model cannot provide a unique solution. Nevertheless, if *Γ*^*(2)*^ is small, the pump pulse is not much affected by the SHG signal. Therefore, neglecting dispersive and phase-modulation effects, the SHG equation shows that *u*_*sh*_ ∝ *Γ*^*(2)*^. Consequently, since in the experiment we measure *P*_*sh*_*|u*_*sh*_*|*^*2*^, we cannot measure the phase of *Γ*^*(2)*^ but only its module. This fact is confirmed by numerically solving Equations (1a) and (1b), from which we find that - for our typical powers - SHG power depends on *|Γ*^*(2)*^*|*, independently on its share between the real and the imaginary parts.

In our experiment, the pump is a TE mode (polarized along *x*), while the SHG signal is a TM mode (polarized along *y*). Therefore, we define the effective second order susceptibility ${\chi }_{eff}^{(2)}$:

5 $$\begin{array}{c}{\chi }_{eff}^{(2)}=|\frac{{{\rm{\Gamma }}}^{(2)}}{K}|,\\ \,K=\sqrt{{A}_{0}}\tfrac{{\int }_{{A}_{\infty }}{E}_{x}^{\ast }({r}_{\perp },{\omega }_{p}){E}_{y}({r}_{\perp },{\omega }_{sh}){E}_{x}^{\ast }({r}_{\perp },{\omega }_{p})dA}{({\int }_{{A}_{\infty }}n{({r}_{\perp },{\omega }_{p})}^{2}|{E}_{x}({r}_{\perp },{\omega }_{p}){|}^{2}dA){({\int }_{{A}_{\infty }}n{({r}_{\perp },{\omega }_{sh})}^{2}|{E}_{y}({r}_{\perp },{\omega }_{sh}){|}^{2}dA)}^{1/2}}.\end{array}$$

${\chi }_{eff}^{(2)}$ is actually the second order susceptibility term if *χ*^*(2)*^ is spatially constant. If not, ${\chi }_{eff}^{(2)}$ is a quantity related to the strength of the second order nonlinearity. *K* is a dimensionless parameter related to the overlap between pump and SH optical modes. If *χ*^*(2)*^ is constant, conversion efficiency between the two optical modes is proportional to *K*. The far *χ*^*(2)*^ is from being constant, the less the SHG efficiency is related to *K*.

### Nonlinear susceptibility estimation

In Table 1 we report *|Γ*^*(2)*^*|*, estimated from the measured SHG power using our model. *|Γ*^*(2)*^*|* is reported for the TE1–TM3 and the TE1–TM5 combinations. We analyzed waveguides of different *L* selected from different positions on the wafer. Moreover, within the same modal combination, we performed measurements on different *w*. The values reported in Table 1 result from this statistical analysis (each value is an average of 64 measurements for the TE1–TM3 combination and 11 measurements for the TE1–TM5 combination). From this estimation we find that *|Γ*^*(2)*^*|* is much stronger for the TE1–TM3 than for the TE1–TM5 combination. The parameters used in the model come from FEM simulations (group indices, modal profiles, propagation constants) and from measurements (refractive indexes, propagation losses). In particular, we use the *α*_*p*_ measured by the cut-back method (8 dB/cm for *w* = 1 µm, 5 dB/cm for *w* = 2.3 µm). Since we cannot measure propagation losses for high order modes, we approximate *α*_*sh*_ ~ *α*_*p*_. In any case, the waveguides considered in this work are very short (between 2 mm and 6 mm), so the effect of a wrong estimation of *α*_*sh*_ is not determinant.

**Table 1:** Estimation of *|Γ* ^*(2)*^*|* from the measurements using the model described in this work.

	Combination TE1–TM3	Combination TE1–TM5
|Γ^(2)^| [fmV^−1^]	1.7 ± 0.2	0.39 ± 0.06
*|K|*	3.8 × 10^−3^	6.4 × 10^−4^
${\chi }_{eff}^{(2)}$ [pmV^−1^]	0.46 ± 0.06	0.6 ± 0.1

Error bars represent one standard deviation of uncertainty and come from the statistical analysis. Calculated values of *|K|* from the modal profiles according to Equation (5). Effective second order nonlinear susceptibility ${\chi }_{eff}^{(2)}$ estimated from *|Γ* ^*(2)*^*|* and *|K|* according to Equation (5). The columns refer to different phase-matching conditions.

Table 1 reports also *|K|* for both combinations. This value is calculated from mode-analysis FEM simulations according to Equation (5). |*K*| is much larger for the TE1–TM3 than for the TE1–TM5 combination, showing that the first is more efficient than the latter for an equal *χ*^*(2)*^ value. This fact is confirmed experimentally, since for the TE1–TM5 combination we need a stronger pump power to generate the same SHG power of the TE1–TM3 combination.

In Table 1 we also give ${\chi }_{eff}^{(2)}$ values, estimated by Equation (5) with the measured *|Γ*^*(2)*^*|* and simulated *|K|* values. Remarkably, the two modal combinations yield comparable ${\chi }_{eff}^{(2)}$ values within the error bars. So, the strong difference in the generation efficiency expressed by *|Γ*^*(2)*^*|* is mainly due to the mode overlap difference, taken into account by *|K|*.

## Role of Strain

### Second harmonic generation under external load

**Figure 3:**
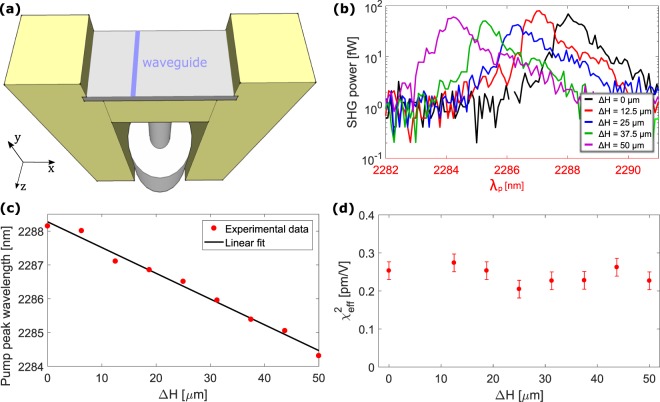
(**a**) Sketch of the screw-equipped sample holder. The total width of the sample is 6 mm, and the analyzed waveguide is located at a distance of about 0.9 mm from the center. (**b**) SHG power (in logarithmic scale) as a function of the pump wavelength for different values of *ΔH* given in the inset. (**c**) Phase-matching wavelength as a function of *∆H*. (**d**) Dependence of ${\chi }_{eff}^{(2)}$ on *∆H*. Error bars come from repeated measurements.

The results presented so far do not offer insights on the origin of the nonlinearity causing SHG. In order to investigate the role of strain, we perform the same experiment described previously using the screw-equipped sample holder sketched in Fig. 3(a)^13,28^. The screw introduces a variable vertical displacement *∆H* (load) in the center of the sample (orthogonally to its main plane), while the sample edges are fixed by the mount. The tensile stress introduced by the SiN cladding (1.25 GPa from wafer bow measurements) sets the initial strain conditions which is then increased by using the screw. A detailed modeling and analysis of this kind of experiments are described in^28^.

Figure 3(b) reports the SHG power as a function of *λ*_*p*_ for different loads applied by the screw. The measurement refers to a waveguide with *w* = 906 nm and *L* = 4 mm, where phase-matching is expected between TE1 and TM3 modes. *∆H* varies from 0 μm (no load) to 50 μm (almost the sample rupture threshold). SHG is observed for all the loads. However, it is noted that the phase-matching wavelength is blue-shifted with increasing strain, while the SHG magnitude does not change significantly.

A linear dependence of the pump phase-matching wavelength as a function of *∆H* is observed (Fig. 3(c)). The maximum phase-matching wavelength shift is about 3.8 nm for *∆H* = 50 μm. This strain-induced shift ensures that we are actually varying the strain inside the waveguide. The shift is due to the waveguide deformation and to the photoelastic effect which cause a strain-induced effective refractive index change for the pump (*δn*_*p*_) and for the SHG (*δn*_*sh*_) modes^28^. The new phase-matching condition requires *n*_*p*_ + *δn*_*p*_ = *n*_*sh*_ + *δn*_*sh*_, determining a modification of the phase-matching wavelength if *δn*_*p*_ ≠ *δn*_*sh*_.

Figure 3(d) shows the dependence of ${\chi }_{eff}^{(2)}$ on *∆H*, where no significant influence on the load increase is observed. This surprising result contradicts the strain as one of the explanations of the observed SHG. Note that the value of ${\chi }_{eff}^{(2)}$ reported in this figure is lower than the mean value of ${\chi }_{eff}^{(2)}$ reported in Table 1. This is due to the large variability of ${\chi }_{eff}^{(2)}$ between different samples, which is taken into account in the statistical analysis reported in Table 1.

### Modelling strain inside the waveguide

**Figure 4:**
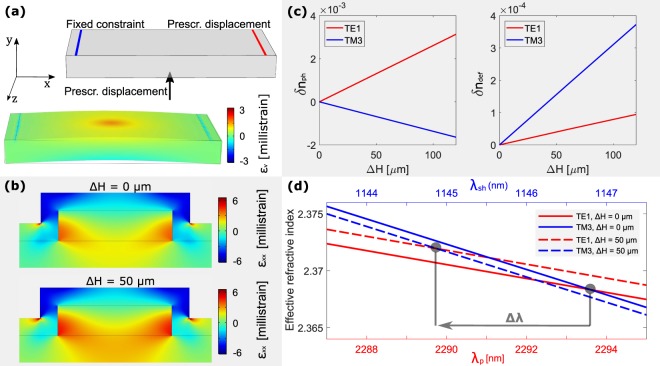
(**a**) On the top: sketch of the simulation domain of the 3D FEM simulation used to estimate the strain introduced by the screw. The supports are modelled by the prescribed displacement and the fixed constraint on the top, while the screw displacement is shown by the arrow. On the bottom: results of the simulation when *∆H* = 50 μm. The volumetric strain *ε*_*v*_ is superimposed in color scale on the deformed sample. Displacements are emphasized by a factor of 10. (**b**) Distribution of the strain tensor element *ε*_*xx*_ inside the waveguide for *∆H* = 0 μm and *∆H* = 50 μm. Simulation refers to a 906 nm wide waveguide. (**c**) Dependence of the effective refractive index variations induced by the photoelastic effect (δ*n*_*ph*_) and by the waveguide deformation (δ*n*_*def*_) on *∆H*. (**d**) Dependence of the effective refractive indexes of the pump (blue line) and SHG (red line) modes on the pump and SHG wavelengths. Simulations are reported for no load (continuous lines) and for *∆H* = 50 μm (dashed lines). Strain induces a shift *Δλ* of the phase-matching condition.

To model the deformation and photoelastic contributions to the change of the phase-matching wavelength, we perform FEM simulations using the procedure described in^28^. First, we perform a 3D simulation of the silicon substrate subjected to the screw load. Elastic parameters of Si, SiO_2_ and SiN are taken from^35–37^. Si and SiO_2_ photoelastic parameters at the pump and SHG wavelengths are interpolated from^38^, while SiN photoelastic parameters are not reported in the literature, so we use the same values of SiO_2_ as we did in^28^. Si parameters are rotated to align to the waveguide crystallographic directions, considering that it is oriented along the [110] direction^35^. Figure 4(a) shows a sketch of the simulation domain, as well as the results of this simulation. A crucial role is played by the screw contact area on the back of the sample. Here we use a screw contact diameter of 7 μm, which yields good agreement with experimental results. We use the results of the 3D simulation as prescribed displacements to perform a local 2D simulation of the waveguide cross-section. Figure 4(b) shows the strain distribution inside the waveguide for *∆H* = 0 μm and for *∆H* = 50 μm, referred to *w* = 906 nm. In the first case, no load is applied, and the waveguide strain is due to the tensile stress introduced by the cladding. In the second case, we consider the maximum load applied by the screw, showing an increase of about 50% of the average strain inside the waveguide. From the simulations reported in Fig. 4(b), we determine two quantities. First, we determine the mean strain value in the waveguide cross-section, from which we estimate the new size of the deformed waveguide. This, in turn, is used to determine the deformation-induced effective refractive index variations *δn*_*p|def*_ and *δn*_*sh|def*_. Second, given the strain distribution in the waveguide and the photoelastic coefficients, we determine the photoelastic-induced effective refractive index variations *δn*_*p|ph*_ and *δn*_*sh|ph*_. We show the *∆H* dependence of these quantities in Fig. 4(c). Deformation introduces an effective refractive index variation for both the TE1 and the TM3 modes. However, this effect is one order of magnitude weaker than the one introduced by photoelasticity. Moreover, photoelastic variation has opposite sign for the two involved modes, due to their different polarization. Considering *δn*_*p*_ = *δn*_*p|def*_ + *δn*_*p|ph*_ and *δn*_*sh*_ = *δn*_*sh|def*_ + *δn*_*sh|ph*_ we compute the phase-matching wavelength as a function of the load. An example is shown in Fig. 4(d). Results refer to *∆H* = 0 μm and *∆H* = 50 μm. Since photoelasticity dominates, the pump effective refractive index increases, while the SHG effective index decreases. This determines a shift of the phase-matching of almost 4 nm, which is comparable with the experiment. Note that the simulated phase-matching wavelength shown in Fig. 4(d) is slightly different from the experimental one shown in Figure 3(b) (2293.8 nm against 2288 nm at *∆H* = 0 μm). This is due to local variations of the waveguide geometry, as already discussed previously. However, what matters is the agreement between the experimental and the simulated strain-induced phase-matching shift.

**Figure 5:**
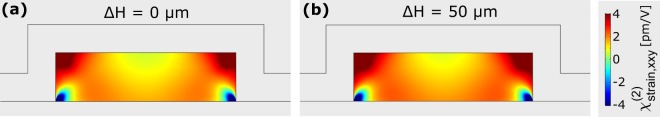
Strain-induced χ^(2)^ map in the waveguide calculated according to the model^16^, and referred to *∆H* = 0 μm (**a**) and *∆H* = 50 μm (**b**).

In^17^, a model to connect the strain gradient components to the strain-induced nonlinear-coefficient ${\chi }_{strain}^{(2)}$ has been introduced. Considering the optical modes involved in the experiment described here, the χ^(2)^ component to be considered is:

6 $${\chi }_{strain,xxy}^{(2)}(\phi )={{\rm{\Gamma }}}_{xxy,xxy}{\eta }_{xxy}(\phi )+{{\rm{\Gamma }}}_{xxy,yyy}{\eta }_{yyy}(\phi )$$

being *η*_*mnl*_ = *dε*_*mn*_*/dl* the strain gradient component, *φ* the orientation of the waveguide with respect to the crystallographic axis (in our case *φ* = 0°), while the coefficients *Γ*_*ijk*,*mnl*_ depend on the nature of the crystal. In^16^, these coefficients are fitted to the experimental data for a Si/SiN electro-optic modulator. Considering *φ* = 0°, they are *Γ*_*xxy*,*xxy*_ = −4 × 10^−16^ m^2^V^-1^ and *Γ*_*xxy*,*yyy*_ = −5.1 × 10^−16^ m^2^V^-1^. By using these values, the ${\chi }_{strain,xxy}^{(2)}$ map inside the waveguide is calculated, and it is shown in Fig. 5 referred to the cases *∆H* = 0 μm and *∆H* = 50 μm. The average ${\chi }_{strain,xxy}^{(2)}$ value varies from around 1.75 pmV^−1^ to about 2 pmV^−1^, showing an increase of about 14%. However, we cannot directly compare these values with the experimental ${\chi }_{eff}^{(2)}$. In fact, the SHG efficiency is not directly related to the ${\chi }_{strain,xxy}^{(2)}$ map, but to the overlap of the ${\chi }_{strain,xxy}^{(2)}$ map with the optical modes. According to Eq. (4), Γ^*(2)*^ can be evaluated using ${\chi }^{(2)}={\chi }_{strain,xxy}^{(2)}$, and ${\chi }_{eff}^{(2)}$ can be consequently estimated using Eq. (5). In the case *∆H* = 0 μm, and considering the modal combination TE1–TM3 described in this work, we estimate ${\chi }_{eff}^{(2)}$ = 0.13 pmV^−1^. In the case *∆H* = 50 μm, ${\chi }_{eff}^{(2)}$ = 0.20 pmV^−1^, more than 50% larger than the value at *∆H* = 0 μm.

To conclude, the model proposed in^16^ applied to our experiment provides a 50% increase of the ${\chi }_{eff}^{(2)}$ as a consequence of the applied load. Surprisingly, in the experiment no significant variation of the SHG efficiency is observed.

## Role of Charged Centers

### Dangling bonds and UV charge passivation

During the deposition of SiN films on the top of silicon, defects can be formed^39,40^. The most significant defect is the *K* center, which is formed by a silicon atom bound to three nitrogen atoms, with a dangling bond. The *K* center has three different charge states: the positive state *K*^+^ (no electrons in the dangling bond), the neutral state *K*^0^ (one electron in the dangling bond) and the negative state *K*^−^ (two electrons in the dangling bond)^23^. The most thermodynamically favorable charge state is the *K*^+^ center. In presence of a Si/SiN interface, *K*^+^ centers form, which results in a positively charged layer at the Si/SiN interface^41^. Going back to the waveguide here analyzed, the positively charged *K*^+^ defects in the cladding introduce a static field *E*_*DC*_ in the waveguide. This field adds up to the optical waves by causing the EFISH process, which can be modelled by an effective second order coefficient ${\chi }_{EFISH}^{(2)}=3{\chi }^{(3)}{E}_{DC}$^7,20^. Even though EFISH is a third order process, it yields SHG as an intrinsic bulk *χ*^*(2)*^.

To remove the influence of EFISH in our measurements, we passivate the charged defects by a 254 nm UV irradiation. Through this mechanism, the positive center *K*^+^ is annihilated and neutralized to the *K*^0^ state^23^. A specific experiment is performed to measure the effect of UV irradiation. A 140 nm thick SiN layer is deposited on a silicon substrate. C-V measurements in a MOS configuration allow estimating the areal charge density σ^42^. We quantify a charge density reduction from σ ~ 1.7 × 10^12^ cm^−2^ to σ ~ 3.1 × 10^9^ cm^−2^ after 23 hours of UV exposure.

### Second harmonic generation after charge passivation

The UV treatment is applied to the strained silicon waveguides. First evidence of the UV treatment is a propagation loss reduction from 8 dB/cm to 4 dB/cm in a 1 μm wide waveguide at a wavelength of 2.3 μm. A similar loss reduction was observed in strip-loaded waveguides, formed by a 27 nm thick SOI patterned by a SiN cladding^42^. Loss reduction was attributed to the neutralization of the SiN charged layer, causing a reduction of the carrier concentration in silicon, and determining a reduced free carrier absorption.

**Figure 6:**
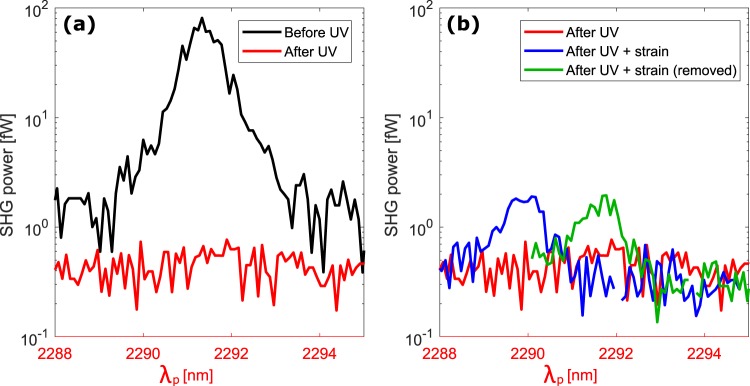
(**a**) SHG power as a function of the pump wavelength before (black line) and after (red line) 23 hours of UV exposure. (**b**) In red: SHG power as a function of the pump wavelength after 23 hours of UV exposure (the same as panel a). In blue: SHG after applying a displacement *∆H* = 25 μm by the screw following the UV exposure. In green: SHG power after removing the screw displacement. The waveguide has *w* = 906 nm.

Figure 6(a) compares the SHG power measured before and after the UV treatment. Notably, a complete suppression of SHG after UV passivation is observed. This indicates that SHG is due to the EFISH process and not to the strain or to the generation in the SiN cladding. As a matter of fact the effect of strain, as well as the generation in the SiN cladding, is below our experimental sensitivity. In our experiments, the noise level sets an upper limit to the nonlinearity of ${\chi }_{eff}^{(2)}$ = 0.05 pm/V, which has to be interpreted as the upper limit of the *χ*^*(2)*^ introduced by strain or by the generation in the cladding. We emphasize that this upper limit is much lower than the strain-induced value calculated here according to the model proposed in^16,17^.

More interestingly, we apply a load to the UV passivated waveguide using the screw-equipped sample holder to verify if a strain-induced *χ*^*(2)*^ can be seen once that the dominant charge contribution is removed. Indeed, the applied strain yields a SHG peak, measured at a pump wavelength position blue-shifted with respect to the original waveguide. This measurement is reported as the blue line in Fig. 6(b). However, when we remove the load, SHG still persists and shifts back to the pump wavelength observed in the original waveguide (green line in Fig. 6(b)). Therefore, since the SHG peak remains and redshifts after the load removal, it cannot be attributed to the applied strain or to a plastic deformation of the sample. We suggest that the observed SHG peak is due to the re-activation of some *K* centers as a consequence of the applied load^43^. In fact, the SHG peak is suppressed by further UV exposition.

### Modelling the effect of charges

To validate our hypotheses on the origin of the SHG, we perform FEM simulations to evaluate the charge-induced field *E*_*DC*_ inside the waveguide. Given the DC field, we estimate the distribution of ${\chi }_{EFISH}^{(2)}$ in the waveguide, and so the parameter *|Γ*^*(2)*^*|*.

In the simulation, we consider an intrinsic p-type doping concentration of 10^15^ cm^−3^ in the waveguide as stated by the wafer supplier for the device layer in the SOI wafer. A positive surface charge density σ = 1.7 × 10^12^ cm^−2^ is applied to the top and to the sidewalls of the waveguide. This value is the one measured for the SiN/Si interface in our LPCVD deposited SiN^42^. For simplicity, in the simulation we assume the charged defects uniformly distributed on the Si/SiN interface, neglecting their extension within silicon nitride. This is however a good approximation, because simulations confirm that the electrical properties within the waveguide are mostly affected by the total charge in the cladding, and not by its distribution.

**Figure 7:**
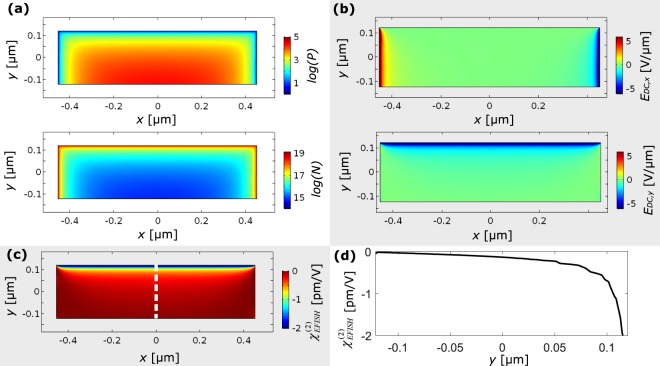
(**a**) Logarithm of the electron concentration *N* and of the hole concentration *P*, expressed in [cm^−3^], when a surface charge density σ = 1.7 × 10^12^ cm^−2^ is present at the side and top borders of the waveguide. (**b**) *x* and *y* components of the DC field inside the waveguide. (**c**) ${\chi }_{EFISH}^{(2)}$ induced by the field inside the waveguide. The value of ${\chi }_{EFISH}^{(2)}$ along the dashed black line is shown in panel (**d**).

Figure 7(a) reports the calculated electron and hole distribution in the waveguide cross section for a *w* = 906 nm waveguide. Simulation shows that the charged defects determine an inversion of the silicon carrier population, with an electron concentration as high as 10^19^ cm^−3^ and a hole concentration as low as 10^5^ cm^−3^. Electrons accumulate close to the positively charged layer, while holes are mainly concentrated near to the uncharged Si/BOX interface. This agrees with previously reported results, where population inversion caused by charges was shown in similar geometries^42,44^.

Given the carrier distribution inside the waveguide, we estimate the carrier-induced absorption using well-known semi-empirical relations^45^. Considering the TE1 mode at the wavelength of 2.3 µm in a *w* = 906 nm waveguide, we estimate a loss reduction of about 4 dB/cm when the charged defects are passivated by UV exposure (σ = 1.7 × 10^12^ cm^−2^ is reduced to σ = 3.1 × 10^9^ cm^−2^). This value agrees with the experiment, where we measure a loss reduction from 8 dB/cm to 4 dB/cm after UV treatment. We ascribe the residual loss of 4 dB/cm to the waveguide sidewall roughnesses.

Using this model, we estimate the electric field *E*_*DC*_ distribution inside the waveguide. In Fig. 7(b) we report the *x* and the *y* components of *E*_*DC*_. They have their maximum value close to the interface, rapidly decreasing when moving into the waveguide.

Knowing the electric field distribution, we compute the SHG efficiency by EFISH. This process relates the pump frequency *ω*_*p*_, the SH frequency *ω*_*sh*_ and the DC field frequency *ω* = 0. Therefore, we need to know the tensor *χ*^*(3)*^*(ω*_*p*_, *ω*_*p*_, *ω*_*sh*_, *0)*. Since literature does not report estimations of it, as a first approximation we use the tensor element measured at the pump frequency *χ*^*(3)*^*(ω*_*p*_,*ω*_*p*_,*ω*_*p*_,*ω*_*p*_*)*. Moreover, in our measurements the pump mode is polarized along *x*, while the SHG mode is directed along *y*. As reported in Fig. 7(b), *E*_*DC*_ has components both along *x* and *y*. Therefore, the tensor elements that can origin the SHG process are the ${\chi }_{xxxy}^{(3)}$ (connected to *E*_*DC*,*x*_) and the ${\chi }_{xxyy}^{(3)}$(connected to *E*_*DC*,*y*_). Because of the cubic symmetry of silicon, ${\chi }_{xxxy}^{(3)}$ = 0^46^. Therefore, only *E*_*DC*,*y*_ plays a role and ${\chi }_{EFISH}^{(2)}=3{\chi }_{xxyy}^{(3)}{E}_{DC,y}$. In the range between 1.2 μm and 2.4 μm, the tensor elements ${\chi }_{xxxx}^{(3)}$ and ${\chi }_{xxyy}^{(3)}$ are related by ${\chi }_{xxyy}^{(3)}={\chi }_{xxxx}^{(3)}/2.36$^47^. However, even the measurements of ${\chi }_{xxxx}^{(3)}$ at pump frequency are ambiguous, ranging from 0.94 × 10^−19^ m^2^ V^−2^ up to 4.24 × 10^−19^ m^2^V^−2^ ^48–50^. Using an average of these values, we obtain the distribution of ${\chi }_{EFISH}^{(2)}$ reported in Fig. 7(c). In Fig. 7(d) we show ${\chi }_{EFISH}^{(2)}$ evaluated on the cutline passing through the center of the waveguide and directed along *y*. A strong ${\chi }_{EFISH}^{(2)}$ is present close to the charged layer, rapidly decreasing as we approach the opposite border of the waveguide. The mean value of ${\chi }_{EFISH}^{(2)}$ in the waveguide is −0.3 pmV^−1^.

Once that the distribution of ${\chi }_{EFISH}^{(2)}$ in the waveguide is known, we evaluate *|Γ*^*(2)*^*|* according to Equation (4) using ${\chi }^{(2)}={\chi }_{EFISH}^{(2)}$. Moreover, we evaluate also ${\chi }_{eff}^{(2)}$ according to its definition in Equation (5). These values are reported in Table 2 and compared with the results evaluated from the experiment. The error bars on the simulated values come from the uncertainty on the tabulated values of ${\chi }_{xxxx}^{(3)}$. For both the modal combinations, simulated values and experimental results are in good agreement considering the simplicity of the model.

**Table 2:** Comparison of the values of *|Γ*^*(2)*^*|* and ${\chi }_{eff}^{(2)}$ estimated from the modelling of the DC field inside the waveguide with results coming from the experiments.

	Combination TE1–TM3		Combination TE1–TM5	
	Experiment	Simulation	Experiment	Simulation
|Γ^(2)^| [fmV^−1^]	1.7 ± 0.2	1.0 ± 0.6	0.39 ± 0.06	0.15 ± 0.10
${\chi }_{eff}^{(2)}$ [pmV^−1^]	0.46 ± 0.06	0.3 ± 0.2	0.6 ± 0.1	0.23 ± 0.15

The error bars on the simulated values come from the uncertainty on the literature reported values of ${\chi }_{xxxx}^{(3)}$.

In this paper, we designed and measured waveguides providing phase-matching on odd parity TM modes. These modes are symmetric along the *x* direction with respect to the waveguide center. In these cases, EFISH is mediated by *E*_*DC*,*y*_, which in turn is symmetric. In this way, the integral at the numerator of Eq. (4) is nonzero. On the other hand, the *E*_*DC*,*x*_ field is antisymmetric with respect to the waveguide center. So, it can enable the generation on even parity modes, which are in turn antisymmetric. Recalling that ${\chi }_{xxxy}^{(3)}=0$, in this case the generation is possible only on TE polarized modes, such as TE4, TE6, and so on. We did not design waveguides providing phase-matching with these combinations, so they are not analyzed in this work.

## Conclusions

In this work, we reported a study of the SHG process in strained silicon waveguide with a SiN cladding. We measured an effective second-order nonlinear susceptibility of about 0.5 pmV^−1^. We devoted particular attention to the investigation of the origin of the measured signal. Studying SHG under external load, we demonstrated no significant dependence of the SHG efficiency value on the applied stress. We then performed a UV treatment of the waveguide to passivate the charged defects formed in the cladding. We showed a complete suppression of the SH signal, demonstrating that charges play a crucial role in the measured phenomenon. Using FEM simulation, we calculated the electric field in the waveguide due to the charged defects and from it the effective second order nonlinearity. A remarkable agreement between measured and computed values is observed, supporting the conclusion that the measured SHG is due to the presence of the charged centers in the cladding. We also verified that the strain effect on *χ*^*(2)*^ is below the noise level, setting an upper limit of 0.05 pmV^−1^ to the strain-induced nonlinear coefficient. The suppression of the SH signal after the UV treatment proves also that a possible generation in the SiN cladding is below the experimental noise. These results settle the origin of SHG in strained silicon, showing that SHG is mainly due to electric field effects caused by the positive charges trapped in the cladding. Interestingly, the upper limit to the measured strain-induced ${\chi }_{eff}^{(2)}$ is comparable with the theoretical results of^51,52^. In these works it was demonstrated that the deformation of the crystalline lattice, induced by the strain gradient, yields low values of *χ*^*(2)*^.

Our outcomes offer interesting perspectives, introducing a paradigmatic change in the development of these kind of structures. Till now great effort was done towards increasing the amount of strain inside the waveguide. However, in this work we evidence that the strain has a secondary role, and large nonlinearities can be obtained by increasing the DC fields inside the waveguide. This can be done both by maximizing the amount of charges deposited on the waveguide border, as well as by realizing thinner waveguides. In fact, Fig. 7(b) shows that the induced field rapidly decreases as far as we move from the charged layer. This clearly has the drawback to increase the propagation losses, since we showed that charges cause the increase of the free carriers inside the waveguide. Therefore, a tradeoff condition must be found between the strength of the DC field and the absorption induced by the carriers.

Furthermore, our work shows that SHG efficiency can be controlled by applying UV irradiation. This offers interesting perspectives for the realization of quasi-phase matched (poled) waveguides. Applying a proper photolithographic mask and exposing it to UV light, periodically varying ${\chi }_{EFISH}^{(2)}$ can be introduced along the waveguide propagation direction. Setting the proper poling period, the conversion between fundamental modes can be directly studied, measuring larger conversion efficiencies due to the stronger mode overlap with respect to the intermodal approach. A recent work already showed the possibility to exploit EFISH in silicon by applying DC fields via lateral p-n junctions^18^. Differently from that work, our suggestion concerns a completely passive device, with no need to apply any external bias.

## References

[1] Lim AEJ (2014). Review of Silicon Photonics Foundry Efforts. IEEE J. Sel. Top. Quantum Electron..

[2] Juan-Colás J (2016). The electrophotonic silicon biosensor. Nat. Commun..

[3] Testa F (2016). Design and Implementation of an Integrated Reconfigurable Silicon Photonics Switch Matrix in IRIS Project. IEEE J. Sel. Top. Quantum Electron..

[4] Borghi M, Castellan C, Signorini S, Trenti A, Pavesi L (2017). Nonlinear silicon photonics. J. Opt..

[5] Liu X (2012). Bridging the mid-infrared-to-telecom gap with silicon nanophotonic spectral translation. Nat. Photonics.

[6] Silverstone JW (2014). On-chip quantum interference between silicon photon-pair sources. Nat. Photonics.

[7] Boyd, R. W. *Nonlinear Optics*. (Elsevier, 2003).

[8] Cazzanelli M, Schilling J (2016). Second order optical nonlinearity in silicon by symmetry breaking. Appl. Phys. Rev..

[9] Jacobsen RS (2006). Strained silicon as a new electro-optic material. Nature.

[10] Chmielak B (2011). Pockels effect based fully integrated, strained silicon electro-optic modulator. Opt. Express.

[11] Damas P (2014). Wavelength dependence of Pockels effect in strained silicon waveguides. Opt. Express.

[12] Azadeh SS, Merget F, Nezhad MP, Witzens J (2015). On the measurement of the Pockels effect in strained silicon. Opt. Lett..

[13] Borghi M (2015). High-frequency electro-optic measurement of strained silicon racetrack resonators. Opt. Lett..

[14] Borghi M (2016). Homodyne Detection of Free Carrier Induced Electro-Optic Modulation in Strained Silicon Resonators. J. Light. Technol..

[15] Olivares I, Angelova T, Sanchis P (2017). On the influence of interface charging dynamics and stressing conditions in strained silicon devices. Sci. Rep..

[16] Berciano M (2018). Fast linear electro-optic effect in a centrosymmetric semiconductor. Commun. Phys..

[17] Damas P, Marris-Morini D, Cassan E, Vivien L (2016). Bond orbital description of the strain-induced second-order optical susceptibility in silicon. Phys. Rev. B.

[18] Timurdogan E, Poulton CV, Byrd MJ, Watts MR (2017). Electric field-induced second-order nonlinear optical effects in silicon waveguides. Nat. Photonics.

[19] Cazzanelli M (2012). Second-harmonic generation in silicon waveguides strained by silicon nitride. Nat. Mater..

[20] Schriever, C. *et al*. Second-Order Optical Nonlinearity in Silicon Waveguides: Inhomogeneous Stress and Interfaces. *Adv*. *Opt*. *Mater*. **3**, 129–136.

[21] Puckett MW (2016). Observation of second-harmonic generation in silicon nitride waveguides through bulk nonlinearities. Opt. Express.

[22] Levy JS, Foster MA, Gaeta AL, Lipson M (2011). Harmonic generation in silicon nitride ring resonators. Opt. Express.

[23] Warren WL, Robertson J, Kanicki J (1993). Si and N dangling bond creation in silicon nitride thin films. Appl. Phys. Lett..

[24] Castellan, C. *et al*. From SHG to mid-infrared SPDC generation in strained silicon waveguides. In *Quantum Photonic Devices* 10358, 1035804 (International Society for Optics and Photonics, 2017).

[25] Trenti, A. *Generation*, *manipulation and detection of NIR and MIR entangled photon pairs*. (University of Trento, 2018).

[26] Osgood RM (2009). Engineering nonlinearities in nanoscale optical systems: physics and applications in dispersion-engineered silicon nanophotonic wires. Adv. Opt. Photonics.

[27] Danielius R (1996). Matching of group velocities by spatial walk-off in collinear three-wave interaction with tilted pulses. Opt. Lett..

[28] Castellan C (2018). Tuning the strain-induced resonance shift in silicon racetrack resonators by their orientation. Opt. Express.

[29] Signorini S (2018). Intermodal four-wave mixing in silicon waveguides. Photonics Res..

[30] *COMSOL Multiphysics*® *v*. *5*.*3a*, www.comsol.com *COMSOL AB*, *Stockholm*, *Sweden*.

[31] Yariv, A. *Quantum electronics*. (Wiley, 1989).

[32] Reed, G. T. & Knights, A. P. *Silicon Photonics: An Introduction*. (Wiley, 2004).

[33] Chang, W. S. *Fundamentals of guided-wave optoelectronic devices*. (Cambridge University Press, 2009).

[34] Agrawal, G. P. *Nonlinear Fiber Optics*. (Academic Press, 2007).

[35] Hopcroft MA, Nix WD, Kenny TW (2010). What is the Young’s Modulus of Silicon?. J. Microelectromechanical Syst..

[36] Ye WN (2005). Birefringence control using stress engineering in silicon-on-insulator (SOI) waveguides. J. Light. Technol..

[37] Edwards RL, Coles G, Sharpe WN (2004). Comparison of tensile and bulge tests for thin-film silicon nitride. Exp. Mech..

[38] Huang M (2003). Stress effects on the performance of optical waveguides. Int. J. Solids Struct..

[39] Robertson J, Powell MJ (1984). Gap states in silicon nitride. Appl. Phys. Lett..

[40] Robertson J, Warren WL, Kanicki J (1995). Nature of the Si and N dangling bonds in silicon nitride. J. Non-Cryst. Solids.

[41] Damas, P. A. L. de S. Pockels effect in strained silicon waveguides. (Université Paris-Saclay, 2016).

[42] Piccoli, G., Bernard, M. & Ghulinyan, M. Permanent mitigation of loss in ultrathin silicon-on-insulator high-Q resonators using ultraviolet light. *Optica* **5**, 1271–1278 (2018).

[43] Pfanner G, Freysoldt C, Neugebauer J, Gerstmann U (2012). Ab initio EPR parameters for dangling-bond defect complexes in silicon: Effect of Jahn-Teller distortion. Phys. Rev. B.

[44] Damas P (2017). Comprehensive description of the electro-optic effects in strained silicon waveguides. J. Appl. Phys..

[45] Nedeljkovic M, Soref R, Mashanovich GZ (2011). Free-Carrier Electrorefraction and Electroabsorption Modulation Predictions for Silicon Over the 1–14-$muhboxm$ Infrared Wavelength Range. IEEE Photonics J..

[46] Rottwitt, K. & Tidemand-Lichtenberg, P. *Nonlinear Optics: Principles and Applications*. (CRC Press, 2014).

[47] Zhang J (2007). Anisotropic nonlinear response of silicon in the near-infrared region. Appl. Phys. Lett..

[48] Hon NK, Soref R, Jalali B (2011). The third-order nonlinear optical coefficients of Si, Ge, and Si 1-x Ge x in the midwave and longwave infrared. J. Appl. Phys..

[49] Bristow AD, Rotenberg N, van Driel HM (2007). Two-photon absorption and Kerr coefficients of silicon for 850–2200 nm. Appl. Phys. Lett..

[50] Lin Q (2007). Dispersion of silicon nonlinearities in the near infrared region. Appl. Phys. Lett..

[51] Hon, N. K., Tsia, K. K., Solli, D. R., Jalali, B. & Khurgin, J. B. Stress-induced χ(2)in silicon — Comparison between theoretical and experimental values. In *2009 6th IEEE International Conference on Group IV Photonics* 232–234, 10.1109/GROUP4.2009.5338380 (2009).

[52] Khurgin JB, Stievater TH, Pruessner MW, Rabinovich WS (2015). On the origin of the second-order nonlinearity in strained Si-SiN structures. JOSA B.

